# First Identification of Novel NDM Carbapenemase, NDM-7, in *Escherichia coli* in France

**DOI:** 10.1371/journal.pone.0061322

**Published:** 2013-04-12

**Authors:** Gaelle Cuzon, Rémy A. Bonnin, Patrice Nordmann

**Affiliations:** Service de Bactériologie-Virologie, Hôpital de Bicêtre, Assistance Publique/Hôpitaux de Paris, INSERM U914, Faculté de Médecine Paris-Sud, France; Institut National de la Recherche Agronomique, France

## Abstract

**Background:**

The NDM-1 carbapenemase has been identified in 2008 in *Enterobacteriaceae*. Since then, several reports have emphasized its rapid dissemination throughout the world. The spread of NDM carbapenemases involve several *bla*_NDM_ gene variants associated with various plasmids among several Gram negative species.

**Methodology:**

A multidrug-resistant *E. coli* isolate recovered from urine of a patient who had travelled to Burma has been characterized genetically and biochemically.

**Principal Findings:**

*E. coli* COU was resistant to all antibiotics tested except amikacin, tigecycline, fosfomycin, and chloramphenicol. Analysis of the antibiotic resistance traits identified a metallo-ß-lactamase, a novel NDM variant, NDM-7. It differs from NDM-4 by a single amino acid substitution sharing an identical extended spectrum profile towards carbapenems. The *bla*_NDM-7_ gene was located on an untypeable conjugative plasmid and associated with a close genetic background similar to those described among the *bla*_NDM-1_ genes. The isolate also harbours *bla*_CTXM-15_ and *bla*_OXA-1_ genes and belonged to ST167.

**Significance:**

This study highlights that spread of NDM producers correspond to spread of multiple *bla*_NDM_ genes and clones and therefore will be difficult to control.

## Introduction

First identified in *K. pneumoniae* and *E. coli* isolates recovered from a patient previously hospitalized in India, NDM carbapenamase producers are of great concern worldwide [1]. NDM carbapenemases are metallo-ß-lactamases that have a very broad substrate profile, including carbapenems, but sparing monobactams. The *bla*_NDM_ genes have been identified mostly in *Enterobacteriaceae* [2], but also in *Acinetobacter* sp. [3], *Pseudomonas* sp. [4], and *Vibrio* sp. [5]. Although NDM-producers have been described worldwide, they are mainly recovered from patients who had a relationship with the Indian subcontinent [2], and in some cases with the Balkan states [6], and the Middle East [7]. Five NDM variants have been described, differing by several amino acid changes. A first variant, NDM-2, has been described in an *A. baumannii* clinical isolate in Germany from a patient native from Egypt [3]. NDM-4, NDM-5 and NDM-6 have been described from *E. coli* isolates from patients with history of hospitalization in India [8]–[10].

NDM carbapenamase carriers are often multidrug-resistant as they co-express other antibiotic resistance genes conferring resistance to ß-lactams (such as other carbapenemase, extended spectrum ß-lactamase, or plasmid-mediated cephalosporinase genes) [2] or to other classes of antibiotics including aminoglycosides (16S rDNA methylases), quinolones and cyclines. These resistance determinants may be carried by identical plasmids and may be co-transferred. Several plasmids harbouring *bla*_NDM_ genes have been sequenced [11]–[13]. Most of them belong to broad host-range plasmids (IncA/C, IncL/M, IncFII, IncH) contributing to the dissemination of the *bla*_NDM_ genes [14].

Unlike other carbapenemase genes, the *bla*_NDM_ genes have been described in *E. coli* that is by far the most frequent community-acquired human pathogen. In addition to India, *E. coli* isolates that produce NDM carbapenemase have also been reported in Canada [15], Cameroon [16], other Asian and European countries [17].

Here, we have analysed an *E. coli* isolate harbouring a novel variant NDM-7 from a patient who had travelled to Burma.

## Results and Discussion

A woman with repeated urinary tract infections travelled to Burma and was then hospitalized in France for surgery of prolapse. Two months later, a carbapenem-resistant *E. coli* COU isolate was recovered from the patient urine culture. Antibiogram determined by the disc diffusion technique and interpreted according to guidelines of the Clinical and Laboratory Standards Institute [18], revealed that *E. coli* COU was resistant to penicillins, expanded-spectrum cephalosporins, and carbapenems. MICs of ß-lactams as shown in Table 1, revealed that *E. coli* COU was resistant to ertapenem, imipenem, and meropenem. It was also resistant to fluoroquinolones, cotrimoxazole, to all tested aminoglycosides except amikacin, and to tetracycline. The isolate remained susceptible only to tigecycline (MIC of 0.38 mg/L), amikacin and chloramphenicol. The presence of a metallo-β-lactamase was assessed by using Etest MBL, which gave a positive result. *E. coli* COU belonged to the sequence type ST167, according to Multi Locus Sequence Typing [19] and to phylogenetic group A. ST167 clone belonged to complex ST10 that has previously been associated with *bla*_CTX-M_ genes in Spain [20].

**Table 1:** MICs of ß-lactams for *E. coli* COU, *E. coli* transformant (*E. coli* DH10B) TfCOU, recombinant plasmids pNDM-7 and pNDM-4 in *E. coli* DH10B and *E. coli* recipient strain DH10B.

	MIC (µg/ml)				
ß-Lactam(s)^a^	*E. coli* COU	TfCOU	*E. coli* pNDM-7	*E. coli* pNDM-4	*E. coli* DH10B
Amoxicillin	>256	>256	>256	>256	4
Amoxicillin+CLA	>256	>256	>256	>256	4
Ticarcillin	>256	>256	>256	>256	2
Ticarcillin+CLA	>256	>256	>256	>256	2
Piperacillin	>256	>256	>256	>256	1
Piperacillin+TZB	>256	>256	>256	>256	1
Cephalothin	>32	>32	nd^b^	nd^b^	4
Cefoxitin	>32	>32	>256	256	2
Cefotaxime	>256	>256	>256	>256	0.06
Ceftazidime	>256	>256	>256	>256	0.06
Aztreonam	>256	0.06	0.06	0.06	0.03
Imipenem	6	4	16	16	0.12
Meropenem	16	3	8	8	0.03
Ertapenem	>32	3	16	16	0.03

a CLA, clavulanic acid at a fixed concentration of 2 µg/ml; TZB, tazobactam at fixed concentration of 4 µg/ml.

b Not determined.

**Figure 1:**
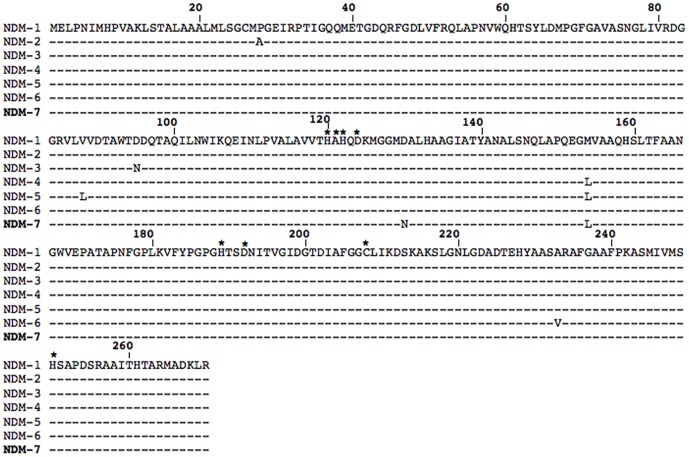
Alignment of the amino acid sequences of the seven NDM variants. Conserved residues of the active site of the metallo-ß-lactamase are denoted with asterisks.

**Figure 2:**
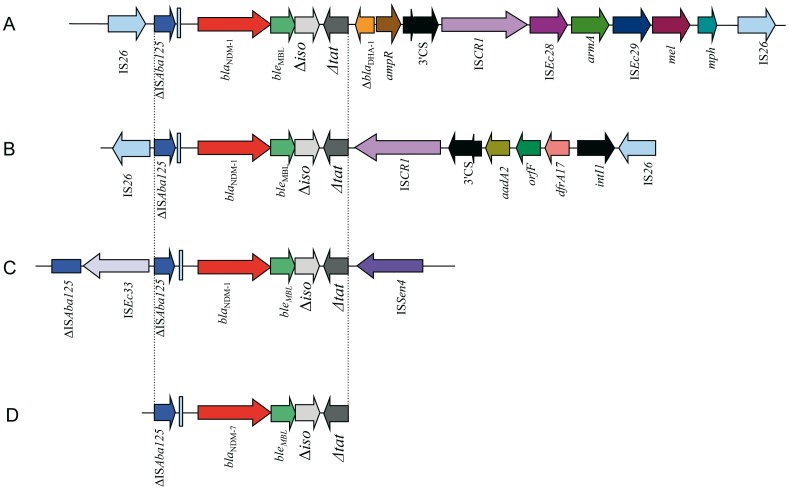
Schematic representation of different genetic structures surrounding *bla*_NDM_ genes identified in *E. coli*. (A) pHKNDM encoding *bla*_NDM-1_ gene described in *E. coli* isolate from Hong-Kong (accession number HQ451074) (11); (B) pGUE-NDM encoding *bla*_NDM-1_ gene described in *E. coli* isolate from India (accession number JQ364967) (12); (C) p271A encoding *bla*_NDM-1_ gene described in *E. coli* isolate from Bangladesh (accession number JF785549) (25); and (D) genetic structure surrounding the new *bla*_NDM-7_ gene from *E. coli* COU. Genes and their transcription orientations are indicated by arrows.

PCR experiments using the whole DNA of *E. coli* COU as template and primers for detection of Ambler class A, class D and class B ß-lactamase genes identified *bla*_NDM_, *bla*_CTX-M_ and *bla*_OXA_ genes. No plasmid-mediated cephalosporinase, 16S rRNA methylase or *qnr* genes could be evidenced, contrary to what has been described for *E. coli* isolates harboring *bla*_NDM_ genes [21]. DNA sequence analysis identified *bla*_NDM-7_ gene (GenBank accession number: JX412225) that had previously been reported once (GenBank accession number: JX262694), *bla*_CTX-M-15_ and *bla*_OXA-1_ genes. NDM-7 differed from NDM-1 by the substitution Met-154-Leu (as in NDM-4) [8], and possessed an additional aspartate-to-asparagine substitution at position 130 (Asp-130-Asn) (Figure 1). Plasmid content of COU isolate revealed three different sized plasmids. A ca. 80-kb plasmid, pCOU, was successfully transferred to *E. coli* Az^R^J53 by conjugation and to *E. coli* DH10B by electroporation. The transformant TfCOU displayed a ß-lactam resistance pattern consistent with the expression of NDM-7 (Table 1). No other antibiotic resistance marker was cotransferred, contrary to what is observed with many plasmids harboring *bla*_NDM_ genes. Plasmid pCOU could not be characterized by PCR-based replicon typing aimed at identifying the main Inc group (FIA, FIB, FIC, HI2, I1-I, L/M, P, N, W, T, A/C, K, B/O, X, Y, F and FIIA) [22]. Upstream of the *bla*_NDM-7_ gene a fragment of insertion sequence IS*Aba125* was found, together with a bleomycin resistance gene downstream of the *bla*_NDM-7_ gene, as previously described for other *bla*_NDM_ genes [12] (Figure 2, panel D). The immediate genetic environment of the *bla*_NDM-7_ gene was identical to that of other *bla*_NDM_ genes identified from Hong-Kong, India, and Bangladesh [11], [12], [25].

NDM-4 has been described with increased hydrolytic activity toward carbapenems [8]. In order to evaluate and compare the spectrum of hydrolysis of NDM-7 and NDM-4 (differing by one amino acid substitution, [figure 1]), the *bla*_NDM-7_ and *bla*_NDM-4_ genes were cloned using ZeroBlunt TOPO PCR cloning kit and then expressed in a same *E. coli* DH10B background. Recombinant strains harboring pNDM-7 and pNDM-4 were resistant or of reduced susceptibility to all ß-lactams except aztreonam. A crude ß-lactamase extract of recombinants strains were obtained and specific activities were determined for ertapenem and imipenem. No significant differences could be evidenced between isolates harbouring either pNDM-7 or pNDM-4 (data not shown). Therefore, the leucine residue at position 154 may explain the common property of extended hydrolytic activity toward carbapenems of both NDM-4 and NDM-7.

This study identified a novel NDM-type ß-lactamase, NDM-7. The strain has been isolated from a patient whose the only link with an endemic country was a travel to Burma. To our best knowledge, *bla*_NDM_ genes have never been reported from this country and this case could further highlights the dissemination of NDM carbapenemases in Southeast Asia, after that in Tahiland [17] and Vietnam [23], [24]. This is also the first description of the *bla*_NDM_ gene in *E. coli* isolate belonging to clonal complex ST-10, a successful clone for spread of *bla*_CTX-M_ genes, as ST-131 clone.

## Methodology

### Bacterial isolates and plasmids

*E. coli* COU was recovered from a urinary culture of a patient. Electrocompetent *E. coli* DH10B (Life Technologies, Saint-Aubin, France) was used as a recipient in electroporation experiments and sodium azide-resistant *E. coli* J53Az^R^ was used as a recipient for conjugation experiments. The ZeroBlunt TOPO PCR vector was used for PCR cloning experiments (Life Technologies) [8]. Natural plasmids were extracted using Kieser extraction method or with Qiagen plasmid DNA maxi kit (Qiagen, Courtaboeuf, France).

### Antimicrobial agents and MIC determinations

Antibiograms were determined by the disc diffusion method and MICs of ß-lactams and tigecycline were determined by Etest (bioMérieux, Marcy-L'Etoile, France) on Mueller-Hinton-Agar (Biorad, Marnes-la-Coquette, France) and interpreted as recommended by the Clinical and Laboratory Standards Institute (CLSI) [18].

### PCR amplification and sequencing

Total DNA from *E. coli* COU isolate was used as template for PCR reactions aimed at searching *bla*_SHV_, *bla*_TEM_, *bla*_CTX-M_, *bla*_KPC_, *bla*_NDM_, *bla*_IMP_, *bla*_VIM_, *bla*_OXA-1/9_, plasmid-mediated cephalosporinase, *qnr* and 16S rRNA methylase genes. Both strands of the PCR products, were sequenced using laboratory-designed primers with an automated sequencer (ABI PRISM 3100; Applied Biosystems). The genetic background of *bla*_NDM_ gene was investigated by PCR mapping and by direct sequencing of pCOU using outward primers. PCR-based replicon typing of the main plasmid incompatibility groups reported in *Enterobacteriaceae* was performed as described [21].

### Multi Locus Sequence Typing

MLST with seven housekeeping genes (*adk*, *fumC, gyrB, icd, mdh, purA and recA*) was performed according to Wirth *et al.* [19]. Allele sequences and sequence types (STs) were checked at the http://mlst.ucc.ie web site.

### Cloning experiments of bla_NDM_ genes

Whole-cell DNAs were extracted as described [8]. The *bla*_NDM_ genes from *E. coli* COU and *E. coli* I5 producing NDM-7 and NDM-4, respectively, were PCR amplified using the *Pfu* thermostable polymerase (Stratagene, Massy, France) and pre-NDM-for and pre-NDM-rev [8], as previously described. These PCR fragments were then cloned into ZeroBlunt TOPO PCR vector (Life technologies), yielding plasmids pNDM-7 and pNDM-4. The sequences of the cloned PCR generated DNA fragments were confirmed by complete resequencing on both strands. Recombinant plasmids were transformed by electroporation into *E. coli* TOP10. Antibiotic-resistant colonies were selected onto Trypticase Soy (TS) agar plates containing imipenem (1 µg/ml).

### Specific activity

ß-Lactamase extracts were obtained as described previously. The specific ß-lactamase activity of the extracts was measured by UV spectrophotometry (spectrophotometer ULTROSPEC 2000, Amersham Pharmacia Biotech, Orsay, France) as described previously [26]. The specific ß-lactamase activities were obtained with 100 µM imipenem and ertapenem as substrates. The total protein content was measured with the Bio-Rad DC protein assay kit (Bio-Rad, Marnes-la-Coquette, France).
